# Edge Computing Based on Convolutional Neural Network for Passenger Counting: A Case Study in Guadalajara, Mexico

**DOI:** 10.3390/s25061695

**Published:** 2025-03-09

**Authors:** Roxana Sánchez Laguna, Ulises Davalos-Guzman, Lina M. Aguilar-Lobo

**Affiliations:** 1 Maestría en Ciencias Computacionales, Universidad Autónoma de Guadalajara. Av. Patria 1201, Zapopan 45129, Mexico; roxana.sanchez@edu.uag.mx (R.S.L.); udavalos2@gmail.com (U.D.-G.); 2 Departamento de Computación e Industrial, Universidad Autónoma de Guadalajara. Av. Patria 1201, Zapopan 45129, Mexico

**Keywords:** edge computing, passenger counting, convolutional neural network, Jetson Nano

## Abstract

One of the most common deficiencies in the public transport system is long waiting times. Currently, in the Guadalajara Metropolitan Area, Mexico, the frequencies of routes are fixed, making it impossible to satisfy a demand with a dynamic variation. An intelligent public transport system is required. The first step to solve this problem is knowing the number of users so that we can respond appropriately to each scenario. In this context, this work focuses on the design and implementation of an embedded system module for passenger counting that can be used to improves public transport service quality. This work presents three contributions. First, a design and experimental validation of the passenger counting system is presented to determine the number of users in an image and send this information to a server suitable for the public transportation system in Guadalajara, Mexico. Second, the generation of two new datasets is reported for training and testing the CSRNet algorithm with images of public transportation systems in Mexican cities. Finally, we make the hardware implementation of the passenger counting system in a Jetson Nano development board.

## 1. Introduction

Public transport is a significant element of urban and economic development, providing individuals access to employment, consumption, health services, education, and recreational activities. For this reason, it is an integral component of the city that allows the population to interconnect the origin–destination of their activities [1]. Any public transport system should maximize the quality of service and reduce travel and waiting times [2]. A well-planned and practical public transportation service is essential because it helps address many environmental issues and reduces traffic congestion in cities [3].

In Guadalajara, Mexico, the public transportation system contributes to 44% of daily transfers. It is made up of two subsystems: the mass transport system and the collective transportation system. The first has three light rail lines and two BRT (Bus Rapid Transit) lines. The second has 233 routes and 5500 units.

However, one of the biggest complaints of users of the mass transport system and the collective transportation system in Guadalajara, Mexico, is the long waiting time due to the saturation of the units or the irregular frequency of their passage. The local authorities fix and approve these frequencies [4]. An analysis of transport demand is performed to define these frequencies using the available information that typically comes from household surveys, transport units, and boarding stations. However, there is no dynamic model to satisfy the variations in demand and reduce the uncertainty of the predicted scenarios [1]. The first step to improve the quality of service of public transportation systems in Guadalajara is to install passenger counting modules that let us know in real time the number of users inside buses or wagons and in the stop-and-go station. With this information, it is possible to build a predictive model to adjust the schedules of the routes in the public transportation systems.

In the literature, several works are related to the people counting for many applications. For example, [5] presents an implementation of a people detection and counting system based on a modified histogram of gradients (HoG), a support vector machine (SVM) using a Field-Programmable Gate Array (FPGA). In another study, a system was designed to calculate the number of people entering and exiting a room. The hardware used was a Raspberry Pi 3 model B, and the information from the number of people is sent to a web server. A VGG-16 was used for feature extraction to detect a person, and the classification was realized using an SVM [6]. An evaluation of different people detection models such as YOLOV4, DetectNet-V2, and Faster-RCNN is presented in Ref. [7], and the hardware implementation of YOLO-V4 was realized using an NVIDIA Jetson Orin.

Many studies about passenger counting systems have been realized using images and video processing techniques. Many works have reported on designing and implementing passenger counting systems using different models and hardware platforms. For example, in Ref. [8], a system was developed using head detection based on Tiny-TOLOv3 on a Samsung ARTIK 710 board, and in Ref. [9], the processing of the WiFi CSI signal in a low-cost X310 device was used as real-time passenger counting system inside a vehicle. In Ref. [10], the deployment of the fine-tuned DL YOLOv8 model is presented over various devices, such as Raspberry Pi4, JEtson AGX ARMv8, JEtson ACG Xavier, Apple M2MAx, Apple MPS, Intel Xeon CPU, Tesla T4, NVIDIA A100, and Testa V100 to evaluate the performance of each platform between devices with a GPU and without a GPU.

Results with a Long Short-Term Memory (LSTM) network as detection algorithm using images [11] or using video was reported in Ref. [12]. For real-time people detection through video, the WatchNET [13] and WatchNEt++ models [14] were presented. In Ref. [15], a model was presented using the CCTV of the buses with the Yolov8 algorithm. The PeopleNet model based on transfer learning was presented in [16], based on VGG16 architecture, and added dense layers by replacing the FC layers with good results.

Concerning the CSRNet algorithm in particular, several works have been reported on crowd counting applications in several scenarios. In Ref. [17], an improved CSRNet architecture was developed to improve the long training and convergence times. Validation was realized using the Shanghai dataset, obtaining similar results with the original CSRNet for high-density images and lower for low-density images, but with training and testing times faster. A model for a dense fish counting network called Swin-CSRNet, which replaced the VGG-16 layer in the front-end by the Swin transformer, was proposed in Ref. [18] for applications in real-world fishery production with better results. To categorize crowds as violent and non-violent, an architecture called VBA (Violent Behavior Analysis) based in CSRNET and Unet was presented in Ref. [19] to identify violent individuals in public events, smart cities, and festivals. Recently, the development of an intelligent passenger counting system for optimizing route networks was presented in Ref. [20]. This work used the YOLOv5 and DeepSORT algorithms for the automated tracking of passenger entries onto and exits off of buses with multiple doors. This work proposed the installation of cameras above each door to maximize coverage and accuracy. As opposed to the work presented in Ref. [20], our work presents the design and validation of an embedded module to collect, process, and transmit information about the number of users of the public transportation system, both inside the vehicles and at stopping stations. The advantage of our embedded module is that it would be installed using the actual infrastructure of the public transportation system in Guadalajara, Mexico, where there is WiFi coverage. Because Guadalajara requires an automated and efficient solution, we decided to use the CSRNet algorithm because it has good performance in passenger counting and assurance of the privacy of the passengers.

The contributions of this work are described as follows:

- Design and validation of a passenger counting system to determine the number of passengers in an image and send the information to a server that can be installed in a public transportation system in Guadalajara, Mexico.
- Two new datasets were generated to train and test the CSRNet algorithm, which was built using images of public transportation systems in Mexican cities.
- Hardware implementation and experimental validation of the passenger counting system with a Jetson Nano developer board.

This paper is organized into four sections. Section 2 presents an overview of the proposed passenger counting system, its components, the hardware and software architecture, and the datasets. Section 3 presents the experimental results and the discussion. Finally, Section 4 concludes the paper.

## 2. Materials and Methods

### 2.1. Passenger Counting System

**Figure 1:**
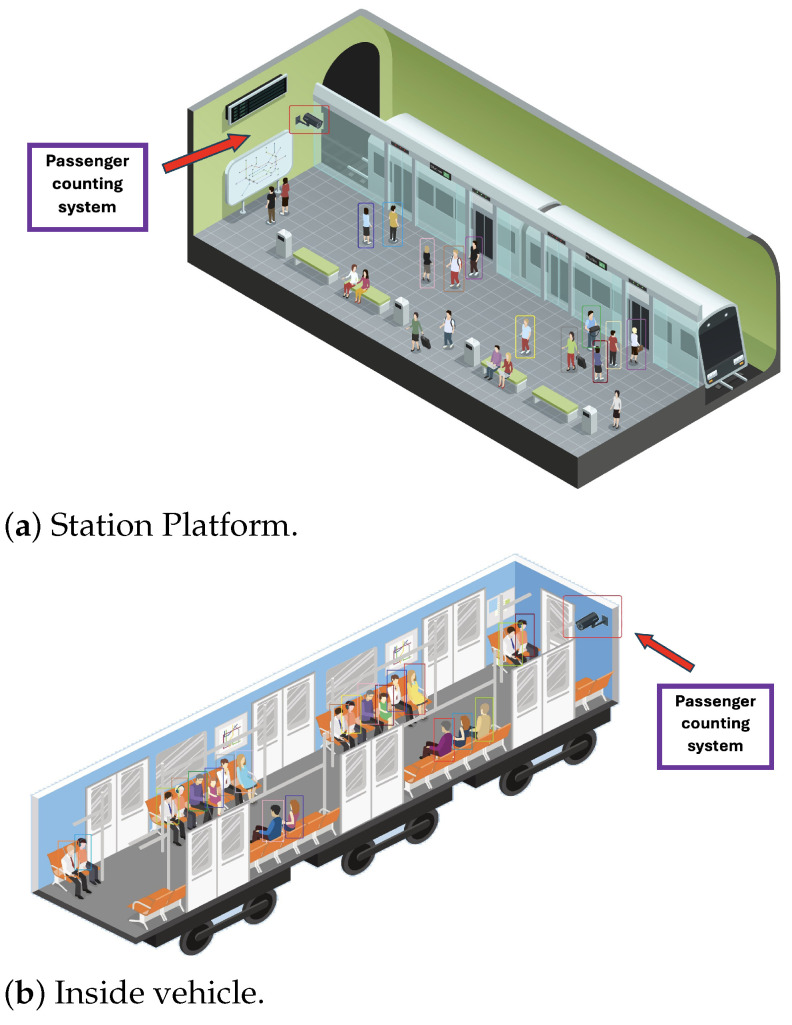
User cases of the passenger counting system.

This work proposed designing a passenger counting system to be used in the public transportation system in Guadalajara, Mexico. The aim is to design a small, lightweight module that could be installed inside vehicles and station platforms in a public transport system. User cases of the passenger counting system are shown in Figure 1.

The passenger counting system development is an embedded module that determines the number of passengers in an image and sends the image information to a server. The principal tasks of the passenger counting system are as follows:

- Capture the images.
- Estimate the number of passengers in the image.
- Save the date and time of the image.
- Save the location of the module.
- Send the above information to a server.

**Figure 2:**
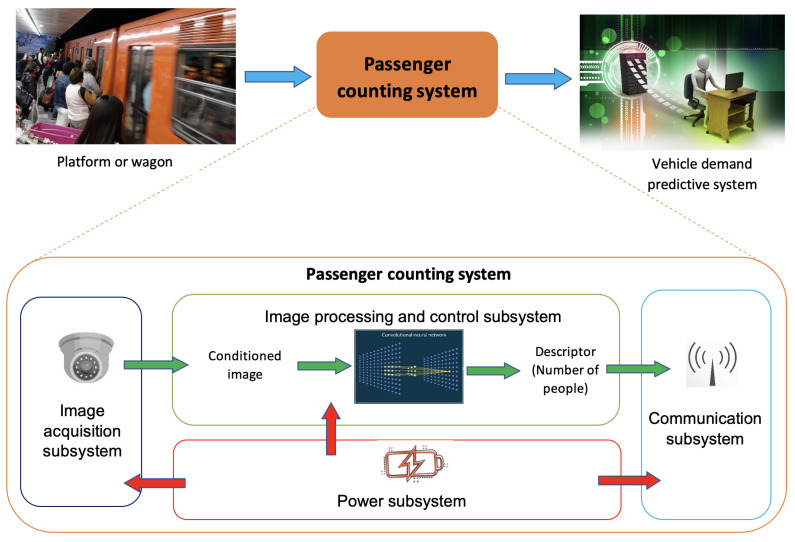
Passenger counting system block diagram.

The components of the passenger counting system are presented in the block diagram of Figure 2:

- Image acquisition subsystem.Its goal is to prepare the input image to make it suitable for the image processing and control subsystem. It is necessary to ensure a precise result when the neural network processes the image.
- Image processing and control subsystem. It is responsible for establishing the connection with the server, managing the image capture, starting the inference, and sending the data to the server periodically. This module is responsible for finishing the execution program and generating the error logs if any processes fail. Its functions are to manage the user interface and the image acquisition subsystem, run the CSRNet algorithm in inference mode to obtain the number of passengers from the image, generate and send information in each inference (number of people, place, date, and time), and record errors produced in any subsystem.
- Communication subsystem. It is based on WiFi to take advantage of the telecommunications infrastructure of all public transport systems in the Guadalajara Metropolitan Area stations. The client–server network model was implemented for this work, with each passenger counting system acting as a client and the Vehicle Demand Predictive System acting as the server.
- Power subsystem. This subsystem must provide electrical power to all other subsystems.

### 2.2. Hardware and Software Architecture

The component used in the image acquisition subsystem is a Spinel camera. This camera has a varifocal lens that allows the field of view to be modified manually. The wide dynamic range function (WDR) allows the viewing of a scene with extreme lighting, that is objects in both illuminated and dark areas [21].

**Figure 3:**
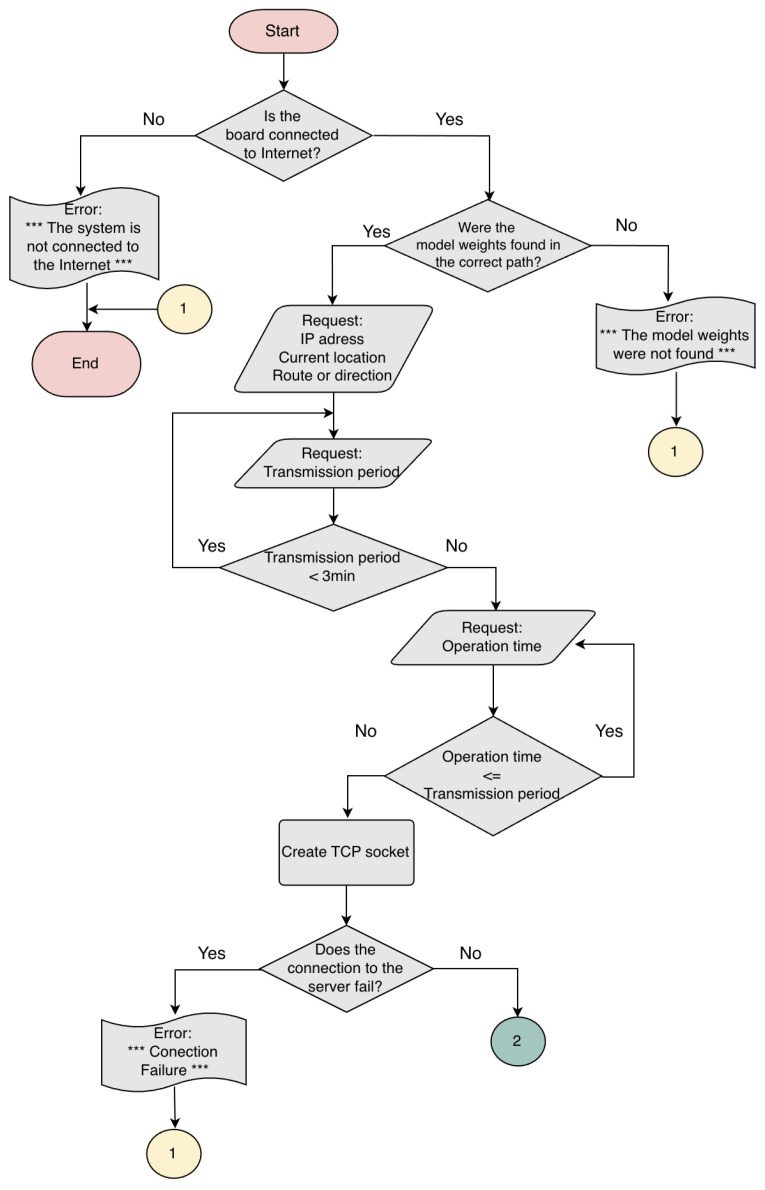
Flowchart.

**Figure 4:**
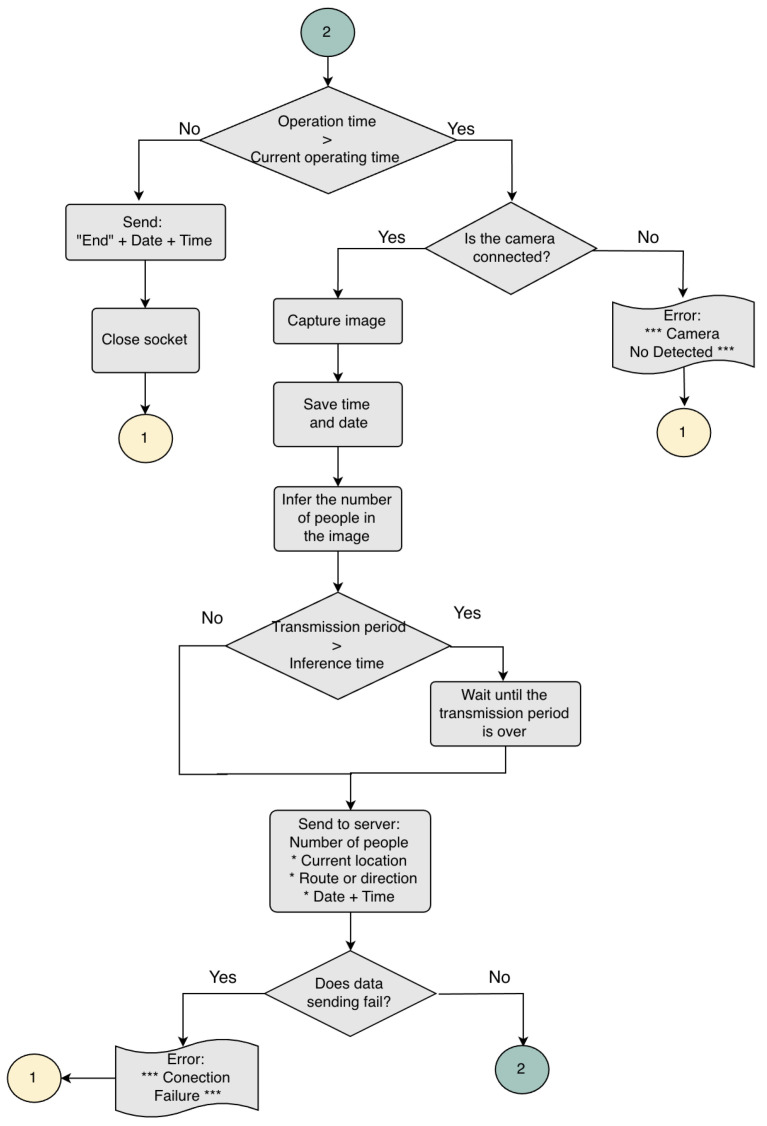
Flowchart.

The image processing and control subsystems need sufficient input/output interfaces to manage all the subsystems, an operating system for hardware management, and high computing capacity for the hardware implementation of the CSRNet model. The Jetson Nano development board [22] was selected for this task. The image processing and control subsystem flowchart is shown in Figure 3 and Figure 4. The data transmission period is greater than the inference time and less than 30 min, and the operation time is established as 18 h.

Because the Jetson Nano does not have a communication interface, an external module was used to transmit the collected information. Due to the infrastructure of all the public transportation systems in Guadalajara having WiFi coverage, we selected this communication technology to transmit the information. The module selected was the Dual Band Wireless AC8265 from Intel. This module can be transmitted in two frequency bands (2.4 GHz and 5 GHz) and is compatible with the IEEE 802.11 standard.

### 2.3. Passenger Counting Model

To obtain the number of people in the passenger counting system, the CSRNet model was used [23]. The CSRNet is based on the CNN VGG-16 to extract high-level features and generate high-quality density maps. The CSRNet model has four different configurations for the back-end, which have the same kernel size and the same number of filters but different dilation rates [24].

#### 2.3.1. Dataset

In Ref. [24], the model was trained and validated with the ShanghaiTech dataset. However, this work does not use this dataset because their images were not taken in any transport system. Therefore, two new datasets with authentic pictures of public transport systems in Mexican cities were created for this investigation. The first dataset is called MxPublicTransport and was used for training and validation. The second dataset is called GdlPublicTransport and was used for model testing. The MxPublicTransport dataset has images with different angles and lighting and represents both scenarios inside the vehicles and in the bus stops on the subway platforms, light rail stations, or BRT stations in Mexican cities. The GdlPublicTransport dataset has images captured from the mass and collective transportation systems in Guadalajara.

The ground truth of these new datasets was realized using manual annotation with a MATLAB (https://www.mathworks.com/products/matlab.html accessed on 4 January 2025) script.

**Figure 5:**
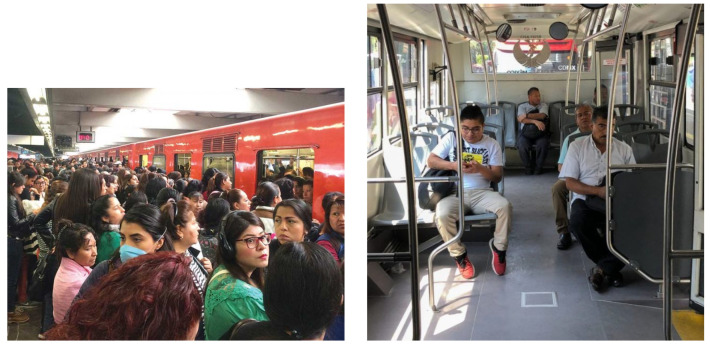
Image examples of the MxPublicTransport dataset in the (**a**) subway platform and (**b**) BRT vehicle.

MxPublicTransport has 712 jpg images randomly downloaded from the Internet with 21,747 labeled people. The MxPublicTransport dataset is divided into a training set with 587 images and a validation set with 125 images. Figure 5a,b show some representative examples of images of the MxPublicTransport dataset. Its passenger density varies between 3 and 94 people.

**Figure 6:**
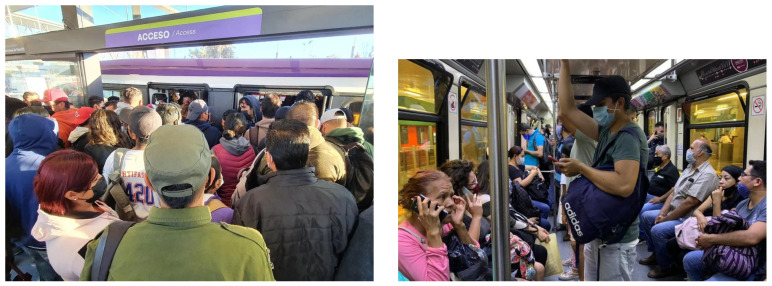
Images examples taken in GdlPublicTransport dataset in a (**a**) BRT platform and (**b**) subway wagon.

The GdlPublicTransport dataset is used for the model testing. It has 166 jpg images from public transport in the Guadalajara Metropolitan Area, downloaded from the Internet, whose dimensions range from 225 × 225 pixels to 3008 × 2005 pixels. The GdlPublicTransport dataset contains 3143 labeled people whose passenger density per image can range from 4 to 44 people. Figure 6a,b present some representative examples of images of the GdlPublicTransport.

#### 2.3.2. Training and Validation

The training and validation with the MxPublicTransport dataset were realized using an environmental configuration provided in Table 1.

Four different configurations of CSRNet were trained and validated to determine the best model for counting people with our dataset. The metrics for evaluating the performance of the four configurations of the CSRNet model are Mean Square Error (MSE), Mean Absolute Error (MAE), the coefficient R2-Score, Mean Absolute Percentage Error (MAPE) [18], and 1-MAPE.

MAE is the mean of the absolute differences between the predicted and actual values, and MSE is the average of the squared differences between the predicted and actual values. Therefore, MSE has no direct interpretation because it has a squared element. MAE only indicates how many people can fail the model in an inference, so it is difficult to visualize the real error percentage. For example, it is not equally relevant to make a mistake of 3 people in an image with 10 individuals, to a mistake of 1 in an image with 10 individuals. For this reason, it is necessary to calculate the MAPE and R2 Score metrics. MAPE is the average of the absolute percentage errors between predicted and actual values. Therefore, it provides the percentage of the prediction error. R2-score measures the correlation between the actual and the predicted values. Therefore, it indicates the precision of the model. Typically, the R2 Score has values between 0 and 1, and values close to 1 indicate that the model explains the data. Additionally, to obtain the percentage of the correctness of the model, we calculate the metric 1-MAPE.

Table 2 summarizes the results for the training process obtained with the MXPublicTransport dataset. It can be seen that the CSRNet Model A has configurations with the better performance. However, differences in the metric values between the four configurations are insignificant since they only vary by a few tenths. Therefore, the CSRNet A was the selected architecture for the implementation of the passenger counting system.

The hyperparameter values of the models used in the training process are detailed in Table 3. Stochastic gradient descent (SGD) was used as an optimizer with a learning rate of $1\times 10^{-7}$. The number of epochs was set to 500. The batch size was 1 because an increase in this negatively impacts the performance of the model.

**Figure 7:**
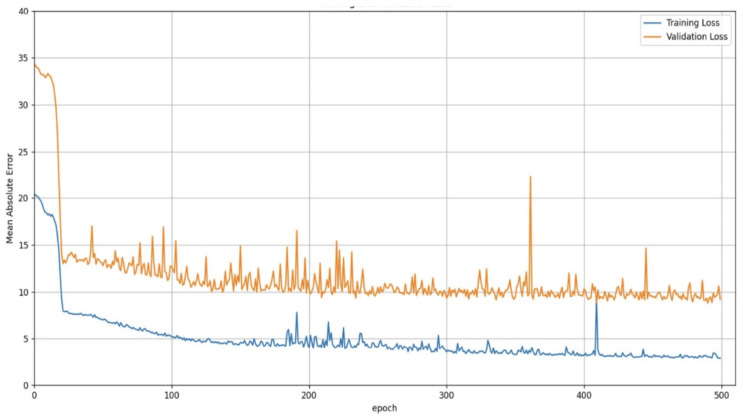
MAE curves on training and validation process for the CSRNet A model with the MXPublicTransport dataset.

**Figure 8:**
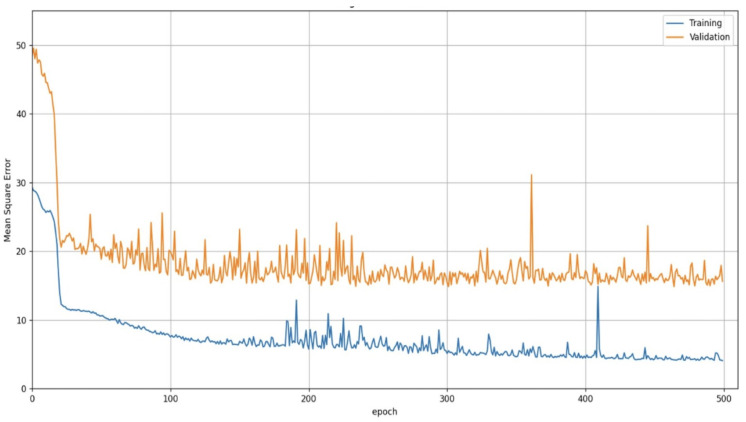
MSE curves on training and validation process or the CSRNet A model with the MXPublicTransport dataset.

**Figure 9:**
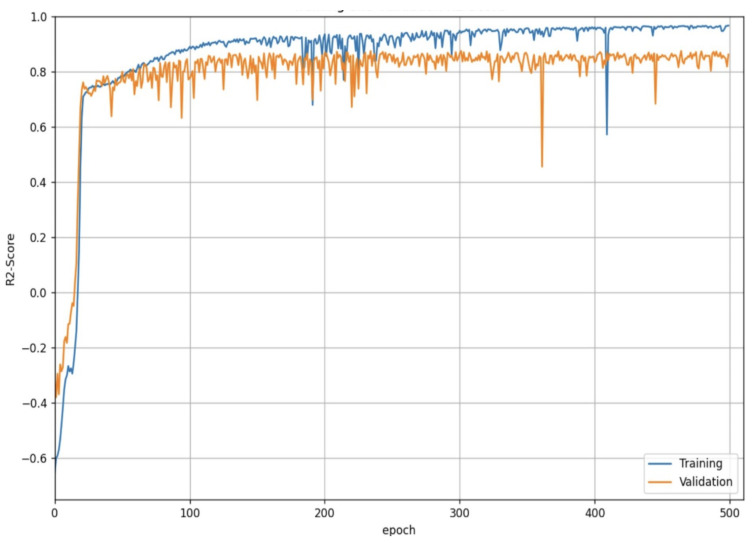
R2-score curves on training and validation process or the CSRNet A model with the MXPublicTransport dataset.

**Figure 10:**
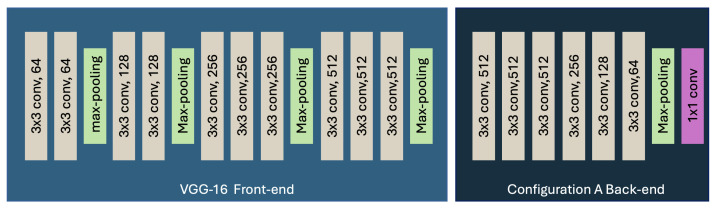
CSRNet-A architecture.

MAE, MSE, and R2-score curves for the training and validation process for the CSRNet A model are shown in Figure 7, Figure 8 and Figure 9. The architecture of the CSRNet A model can be seen in Figure 10.

The testing process for the CSRNet A model was realized using two datasets. Table 4 summarizes the results. As for the training and validation process, we can see that the model with the better performance is the CSRNet A model.

## 3. Experimental Results and Discussion

**Figure 11:**
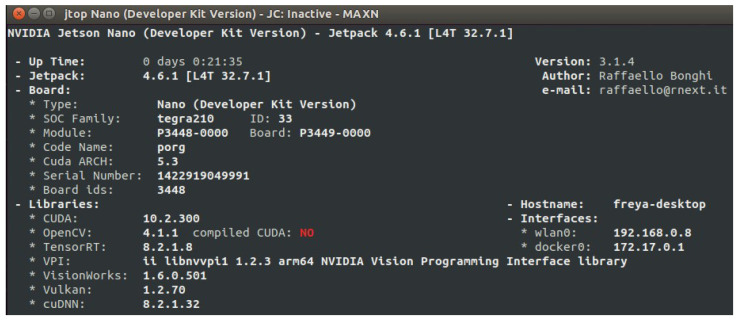
Features of the NVIDIA JetPack SDK.

To validate the effectiveness of this work, we carried out an experimental validation in a laboratory environment. The hardware implementation of the passenger counting system was executed in the Jetson Nano developer board. The Jetson Nano development board was selected as the image processing and control subsystem. Its memory capacity is sufficient for the model and the Docker container, because the CSRNet A model trained weighs 2 MB and the container weighs 1.2 GB. The NVIDIA JetPack SDK [25] is adequate for the implementation because it supports the PyTorch (https://pytorch.org/ accessed on 4 January 2025) and CUDA libraries necessary to run the CSRNet model. In Figure 11, the parameters of the NVIDIA Jetson Nano board developer kit version jetpack 4.6.1 are presented.

The following experiments were carried out:

- Implementation and comparison of results in the PC with the environmental configuration of Table 1 and in the Jetson Nano developer board (Table 5) using the GdlPublicTransport-64, which is composed by 64 images of the GdlPublicTransport, specifically those that are smaller in size due of the constraint of the RAM in the embedded system.
- Implementation of the LightRail-BRTMacroPeriferico dataset in the Jetson Nano. This dataset comprises 16 images with dimensions of 320 × 240 pixels that were captured inside the public transport systems in Guadalajara, Mexico.

For the inference, the jetson-inference container was cloned [26], which has PyTorch pre-installed in a Python3 environment. The Docker container is 1.2 GB in size. Considering that the minimum size recommended by NVIDIA for the microSD card is 32 GB, the embedded software would only occupy less than 10% of the memory capacity. Table 5 presents the technical characteristics of the Jetson Nano developer board.

Table 6 presents the performance metrics results for the experimental results for the inference in the hardware implementation. The results obtained of the inference in the PC and the Jetson Nano, with the GdlpublicTransport-64 dataset, have similar values; they show a good performance of the model in the hardware. However, when the LightRailBRTMacroPeriferico is used, the metric presents a performance drop. This may be due to the images captured in the public transportation systems, principally due to illumination conditions. However, our results are better that the metrics reported in other people counting research [16]. It can see in Table 7 the error magnitude between the ground truth and the hardware implementation for a representative sample of the LightRailBRTMacroPeriferico dataset. In some cases, the number of predicted values is significantly less than the actual value.

**Figure 12:**
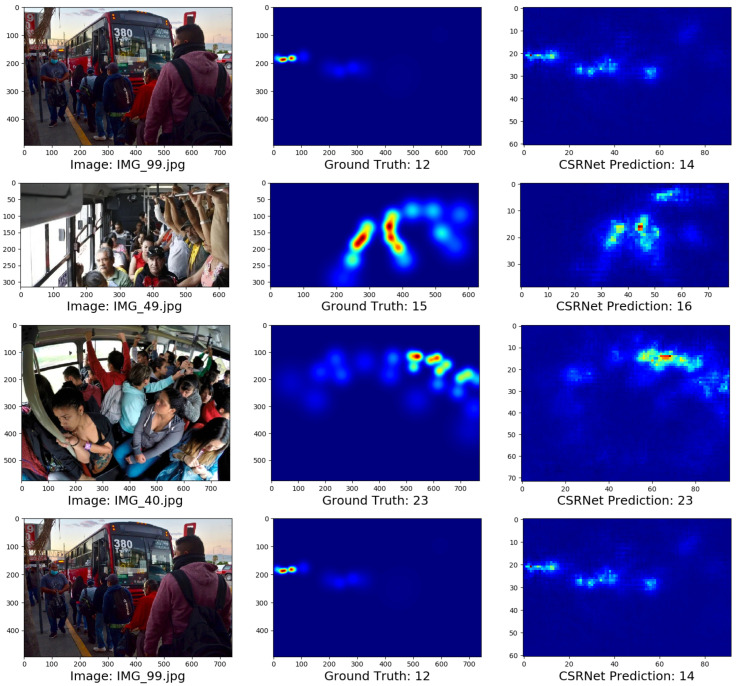
Comparative between the ground truth and the hardware implementation results of the experiments described in Figure 12.

Comparisons between the density maps and the prediction results of the hardware implementation of some images are described in Figure 12. The images show that the prediction results are very close to the ground truth values. These results reveal that our model performs well and can be used in an embedded module as a passenger counting system.

### Parameters of The Prototype

We defined the data transmission frequency as three minutes because a waiting time is given to allow people to accumulate in the area. This interval time is sufficient to generate the capture and the inference between images. This time may vary depending on the requirements of each station or vehicle. However, this time cannot be less than the minimum period, which is defined by the time required to capture the image, the inference time, and the transmission time. The time inference obtained by the passenger counting system in the hardware implementation is between 2 and 130 s.

Additionally, the working time of the passenger counting system must be defined, and it was set at a maximum of 20 h. We set this parameter at 18 h each day for the experimental validation.

When the system boots, it is necessary to set the working time and information about the location, which must indicate the station, direction, or platform where the passenger counting system is installed.

**Figure 13:**
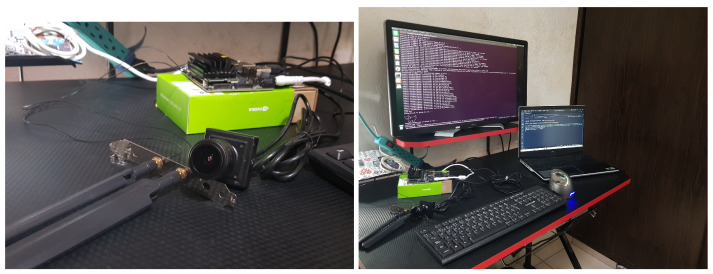
Prototype of passenger counting system.

The final prototype is presented in Figure 13. A computer was used as the system server.

**Figure 14:**
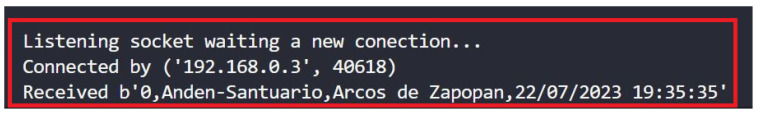
Message received by the server.

The information sent by the passenger counting system to the server has the following format:

- Passenger. Number of passengers in the image.
- Location. The place where the image was taken. The message can be in two different formats. When the image is taken in a station, the message contains information about the station. When the images are taken inside a vehicle, the message contains information about the wagon/vehicle number and departure station. Figure 14 has an example of the received message by the server.
- Route. Arrival station name.
- Date. Format dd-mm-yyyy
- Time hh:mm:ss

The main contributions of this work are the design and validation of a passenger counting system to determine the number of passengers in an image and send this information to a server, the generation of two new datasets, and the hardware implementation of the systems.

**Figure 15:**
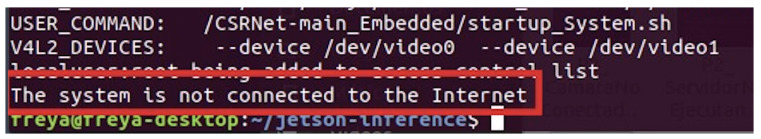
Message received by the server.

The results about the precision of the image processing show that the CSRNet model was correctly trained to recognize and count people in images of the public transportation systems in Guadalajara, Mexico. In some results, there is a difference between the number of people in the image and the number of people inferred by the model; this may be due to lighting conditions when capturing the images. However, the results demonstrate that the developed system could be used within the public transportation system both on the platforms and within the units. The control and data transmission results show that the passenger counting system has a good inference time of one minute and a minimum frequency transmission of three minutes. These times are suitable for analyzing the mobility and number of people using a public transportation system. The prototype validation demonstrates that the system could operate during the total daily operation of a public transportation system in Guadalajara, which is approximately 20 h. The validation of the prototype in a laboratory environment shows that the information about the number of passengers in a vehicle or a platform, the location of the wagon/vehicle or departure station, the arrival station name, and the date and the time when the image was taken, is sent correctly to the server. Moreover, the embedded module has the advantage that it sends an error message when it loses the WiFi connection during the working operation, when it is not possible to connect to the server, or when the camera has not been detected, and saves this error message in a text log (see Figure 15). These results demonstrate that our system can be used to know the number of users inside buses or wagons and in the stopping station in the public transportation systems in Guadalajara. With this information, it is possible to develop a predictive model to adjust the schedules of the routes.

## 4. Conclusions

This work presents an edge computing model based on the CNN for passenger counting that is appropriate for Guadalajara in Mexico. Our solution is based on an embedded module to collect, process, and transmit information about the number of users in the public transportation system. Our design has the advantage that it would be installed using the actual infrastructure of the public transportation system in Guadalajara, Mexico. The embedded model developed uses the CSRNet model for the passenger counting task, which was trained using our datasets built with images of Mexican public transportation systems. Using this model, we assure the privacy of the passengers. The validation shows that the model of our passenger counting system performed well. During the laboratory environment validations, we evaluated the precision of the model in the hardware implementation, the inference time, the transmission and operation time, and the reception by the server. We have validated that the information received for the service is correct. The next step is to build a real prototype and validate its performance in a real environment. Moreover, we will build a new dataset to expand the current dataset and improve the model metrics.

## Figures and Tables

**Table 1:** Environmental configuration of PC used for the experiments.

Configuration	Model/Version
CPU	12 th Gen Intel Core i7-12700KF 3.61 GHz
GPU	NVIDIA GeForce RTX3070
Running Memory	16 GB
Operation System (OS)	Windows 11
Computer Programming Language	Python 3.11.4
Accelerated Environment	CUDA 12.2
Deep Learning Frameworks	Pythorch 2.0.1

**Table 2:** Performance metrics for different CSRNet configurations for training and validation process with MXPublicTransport dataset.

	Training			Validation		
Model	MAE	MSE	R2 Score	MAE	MSE	R2 Score
CSRNet A	2.88	4.08	0.96	9.21	15.61	0.86
CSRNet B	3.03	4.29	0.96	9.49	15.43	0.86
CSRNet C	3.29	4.89	0.95	10.00	16.53	0.84
CSRNet D	2.93	4.12	0.96	9.16	15.19	0.87

**Table 3:** Hyperparameters of the CSRNet models.

Hyperparameter	Value
Optimizer	SGD
Learning rate	$1\times 10^{-7}$
Epochs	500
Batch size	1
Momentum	0.95

**Table 4:** Performance metrics for the four CSRNet configurations for the testing process with the GdlPublicTransport dataset.

Dataset	Number of Images	MAE	MSE	R2 Score	MAPE	1-MAPE
GdlPublicTransport-166	166	3.24	4.16	0.78	0.24	0.78
GdlPublicTransport-64	64	2.61	3.23	0.73	0.17	0.82

**Table 5:** Technical characteristics of the Jetson Nano developer board [22].

Characteristic	Description
CPU	Quad-Core Arm Cortex-A57
GPU	128-core NVIDIA MAxwell
Memory	4 GB 64-bit LPDDR4 25.6 GB/s
Storage	16 GB eMMC 5.1
Operating System	Jetson Linux 34.1.1 (Ubuntu)
Connectivity	10/100/1000 BASE-T Ethernet
Computer Programming Language	Python 3.6
Accelerated Environment	CUDA 10.2.300 cuDNN 8.2.1.32
Deep Learning Frameworks	Torch 1.10.0

**Table 6:** Experimental results of hardware implementation.

Environment	Datasets	Images	MAE	MSE	MAPE	1-MAPE
PC	GdlPublicTransport-64	64	2.61	3.23	0.17	0.82
Jetson Nano	GdlPublicTransport-64	64	3.17	7.17	0.20	0.79
Jetson Nano	LightRailBRTMacroPeriferico	16	4.5	13.06	0.35	0.65

**Table 7:** Error magnitude between the ground truth and the hardware implementation for a representative sample of the LightRailBRTMacroPeriferico dataset.

Image	Ground Truth	Prediction	Error
IMG100	12	10	2
IMA101	8	6	2
IMA105	12	12	0
IMA168	27	22	5
IMA108	10	7	3
IMA116	15	13	2
IMA121	13	10	3
IMA115	15	9	6
IMA127	14	10	4
IMA128	20	22	2
IMA139	13	12	1
IMA149	9	8	1
IMA181	25	15	7
IMA116	15	13	2
IMA88	22	16	6
IMA49	15	15	0

## Data Availability

The data that support the findings of this study are available from the corresponding author upon reasonable request.

## Abbreviations

The following abbreviations are used in this manuscript:

HoG	Histogram of Oriented Gradients
SVM	Support Vector Machine
VGG	Visual Geometry Group
Adaboost	Adaptive Boosting
YOLOV	You Only Look Once
FPGA	Field-Programmable Gate Array
CNN	Convolutional Neuronal Network
ML	Machine Learning
CSRNet	Congested Scene Recognition Net
RPC	Radar-based People Counting
WDR	Wide Dynamic Range
BRT	Bus Rapid Transit
MAPE	Mean Absolute Percentage Error
MSE	Mean Square Error
MAE	Mean Absolute Error
